# Surveillance of Esophageal Cancer in the Republic of Uzbekistan from 2000 to 2018

**DOI:** 10.31557/APJCP.2020.21.8.2281

**Published:** 2020-08

**Authors:** Abror Yusupbekov, Mitsuro Kanda, Bekhzod Usmanov, Otabek Tuychiev, Sayfiddin Baymakov, Junichi Sakamoto, Akhrorbek Yusupbekov

**Affiliations:** 1 *Republican Specialized Scientific and Practical Medical Center of Oncology and Radiology (RSSPMCO&R), Tashkent, Uzbekistan.*; 2 *Department of Gastroenterological Surgery (Surgery II), Nagoya University, Graduate School of Medicine, Nagoya, Japan. *; 3 *Surgical Department, Tashkent State Dental Institute, Tashkent, Uzbekistan. *; 4 *Tokai Central Hospital, Kakamigahara, Japan. *

**Keywords:** Esophageal cancer, incidence, dynamics, Republic of Uzbekistan

## Abstract

**Background:**

Differences in the geographical distributions of esophageal cancer (EC) are associated with environmental influences and genetic risk factors. The inhabitants of the Republic of Uzbekistan are at high-risk for EC; however, detailed epidemiological data regarding the dynamics of EC are not available.

**Methods:**

To address this gap in our knowledge, here we reviewed trends in the incidence of EC in Uzbekistan from 2000 through 2018. We acquired the epidemiological data for 17,144 patients with EC from the national epidemiological data base of Uzbekistan.

**Results:**

The mean incidence (per 100,000 persons) during the study period was 2.8, which peaked at 3.9 in 2007 and decreased below 2.5 in 2014 and thereafter. The incidence was highest for patients aged 61 years to 70 years (37.5%). Among patients with EC, 13,331 (80.0%) and 3,333 (20.0%) were diagnosed with squamous cell carcinoma or adenocarcinoma, respectively. The incidences of patients with EC with adenocarcinoma were 0.6 from 2010–2018 and 0.4 from 2000 to 2009. The majority of patients were diagnosed with stage III EC, which was associated with a 5-year survival rate that increased from approximately 15% (2000–2009) and plateaued at approximately 25% (2012–2018).

**Conclusions:**

We conclude that preventing the progression of EC to stage III is required to improve the prognosis of patients with EC who reside in Uzbekistan.

## Introduction

Esophageal cancer (EC) is the ninth most frequent malignancy worldwide (572,034 registered cases) and has the sixth highest mortality rate (508,585 per year) (Bray et al., 2018). Most of the cases of EC (>80%) were diagnosed in developing countries in 2008, and the geographical incidence of EC varies approximately 20-fold (Di Pardo et al., 2016; Hamrah et al., 2014). EC develops more frequently in men (70%) than women, persons aged more than 70 years represent approximately 40% of cases, and the highest incidence includes those aged 50–60 years (Lagergren et al., 2017). However, these frequencies vary according to geographical regions, which are associated with different exogenous and endogenous factors such as ethnicity (Domper Arnal et al., 2015; Kanda et al., 2019a; Nassri et al., 2018). High-risk geographical areas extend from Northern Iran through Turkmenistan, Northern Afghanistan, Uzbekistan, and Kazakhstan to Northern China. Thus, this region is called the Asian esophageal cancer belt (Asombang et al., 2016; Marjani et al., 2010; Zhang et al., 2012). 

Decades of investigations revealed that risks at development of EC depends on local habits and environmental exposures (Murphy et al., 2017; Sheikh et al., 2019). In contrast to Europe and America, where alcohol consumption and smoking are the most important risk factors for EC, the causes of the high incidence rates of esophageal cancer in developing countries of Central Asia are due to a combination of factors, including thermal injury (from hot tea), exposure to polycyclic aromatic hydrocarbons (from opium and indoor air pollution), unpiped water, tooth loss and nutrient-deficient diets (Lagergren et al, 2017; Sheikh et al, 2019).

Republic of Uzbekistan is a country in Central Asia, and is geographically close to Afghanistan and Golestan province in northeastern Iran where has long been known to have one of the highest reported rates of EC (Rahmani et al., 2018; Sheikh et al, 2019). Hamrah et al., (2014) and (2017) reported that Uzbek-Turkmen ethnic group had a high incidence of esophageal cancer in the northern part of Afghanistan and opium use, nass use and hot tea consumption were significantly more common in the Uzbek-Turkmen patients with EC compared with other ethnic groups.

Although inhabitants of Uzbekistan are at high risk of EC, detailed information related to the dynamics of EC are unavailable. The aim of the present study therefore was to review the trends in the incidence of EC in Uzbekistan from 2000 through 2018.

## Materials and Methods

All data was obtained from 17,144 patients who were registered in the National Cancer Registry Database of the Republic of Uzbekistan, which is a freely-available open-access registry system, from 2000 through 2018 (accounting form No. 7-MH). There are some subsections of the National Cancer Registry Database including reports from the State Committee of Statistics of Uzbekistan for epidemiological information and the Report on the Incidence of Malignant Neoplasms for oncological data. The State Committee of Statistics of Uzbekistan is the organization, objective of which is doing whole statistics in all fields of the Uzbekistan (including demographic statistics). The Report on the Incidence of Malignant Neoplasms includes all patients with EC registered not only in designated hospitals, but also in any other medical stations, and is annually issued by the committee of the National Cancer Registry Database of the Republic of Uzbekistan. The 5-year survival rates were calculated based on the follow-up data from the Report on the Incidence of Malignant Neoplasms. Staging was performed according to the guidelines of the American Joint Committee on Cancer Staging Manual (7th edition) (Zhang et al., 2017). Data for smoking history, localization of primary EC lesions, and treatment profiles were collected from medical records of patients who underwent surgery at the Department of Thoracic Oncology, Republican Specialized Scientific and the Practical Medical Center of Oncology and Radiology (RSSPMCO&R), Tashkent, Uzbekistan, and all of those had been registered in the National Cancer Registry Database of the Republic of Uzbekistan.

## Results

*Epidemiological characteristics of patients residing in Uzbekistan*

**Figure 1:**
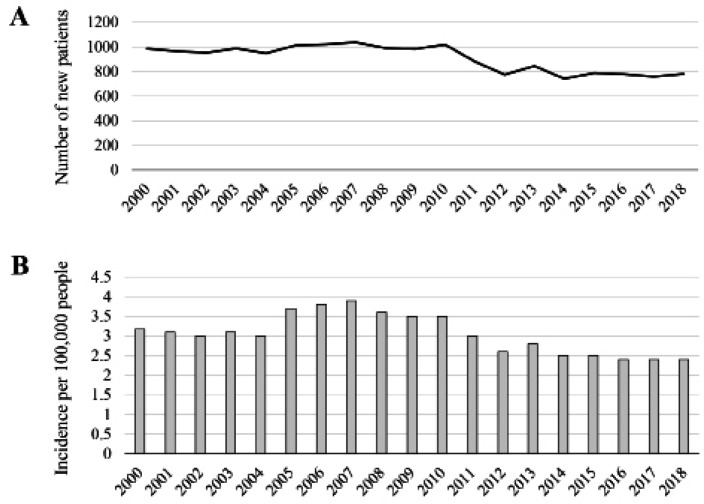
Epidemiology of Esophageal Cancer in Uzbekistan. Yearly variations in the numbers of new patients with esophageal cancer (A) and incidence per 100,000 persons (B).

**Figure 2:**
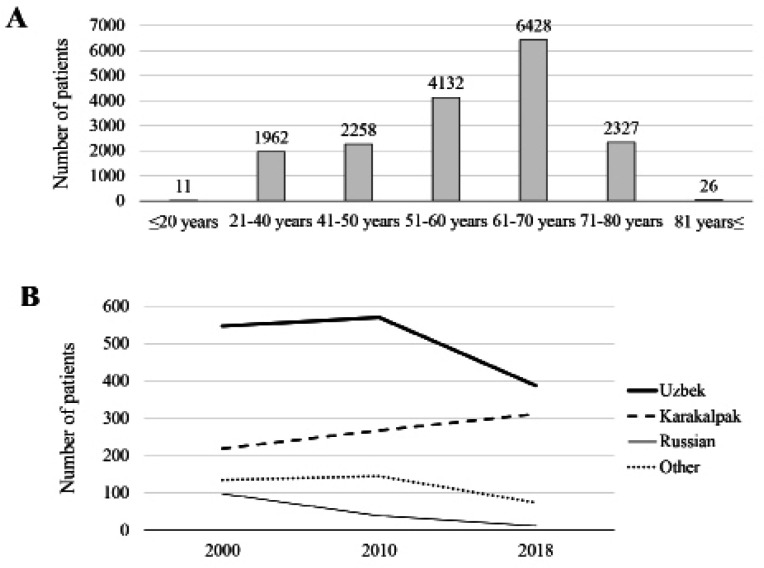
Age and Ethnicity. (A) Distribution of age upon diagnosis. (B) Prevalence of ethnicities of patients diagnosed in 2000, 2010, and 2018

We included 17,144 patients who were treated for EC from 2000 through 2018 (Figure 1A). Approximately 1,000 patients were registered each year from 2000 through 2010, with a gradual decline thereafter to 800 each year (Figure 1A). The mean incidence (per 100,000 persons) of EC was 2.8 during the entire study period, peaking at 3.9 in 2007, declining to 2.5 in 2014, and remaining essentially constant thereafter (Figure 1B). The age distribution upon diagnosis is presented in Figure 2A. Seventy five percent of patients were included in the 51–80 year group. The highest percentage of patients (37.5%) included those aged 61 years to 70 years, and fewer patients were aged less than 20 years or more than 81 years. Ethnicity data were available for 2,797 patients diagnosed in 2000, 2010, and 2018. The most common ethnic groups were, in order of descending prevalence, Uzbek, Karakalpak, and Russian (Figure 2B). The proportion of Karakalpaks linearly increased during the study and that of Russians gradually decreased. Moreover, the proportions of Uzbeks and “others” significantly decreased starting in 2010.

*Histological types of EC*

The histological types of EC of 16,664 patients (97.2%) were diagnosed as squamous cell carcinoma (n = 13,331 [80.0%]) and adenocarcinoma (n = 3,333 [ 20.0%]). In comparison, the prevalence (per 100,000) between early (2000–2009) and late (2010–2018) decreased to 1.8 from 2.4, whereas that of adenocarcinoma increased to 0.6 from 0.4.

*Disease stages upon diagnosis and 5-year survival rates*

**Figure 3:**
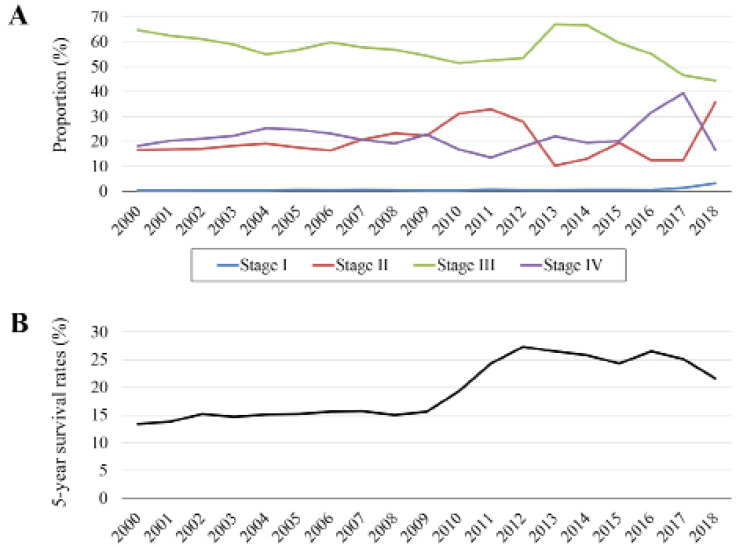
Disease Stage and Prognosis. (A) Annual variations in disease stages upon diagnosis. (B) Five-year survival rates of patients with esophageal cancer

The yearly transitions of disease stages upon diagnosis are shown in Figure 3A. Relatively few patients were diagnosed with stage I EC during most of the study period, with a gradual increase after 2016. In contrast, stage III EC was most frequent, though it exhibited a declining trend after 2014. The proportions of stages II and IV EC were similar. From 2000 through 2009, the 5-year survival rate varied by approximately 15% (Figure 3B), gradually increased to 25%, and remained nearly constant after 2012.

*Clinical characteristics of patients treated at the Republican Specialized Scientific and the Practical Medical Center of Oncology and Radiology (RSSPMCO&R)*

Among the 17,144 registered patients, 8,007 (46.7%) were treated at the RSSPMCO&R. These patients included 4,587 (57.3%) men and 3,420 women (42.7%), ranging in age from 17 years to 82 years (median 63 years). The median (range) ages of men and women were 62.5 (17–82) years and 64 (22–77) years, respectively. Among the 8,007 patients, 1,127 (14.1%) underwent surgery at the Department of Thoracic Oncology of the RSSPMCO&R. Patients’ clinical characteristics were as follows: 519 (46.1%) patients had a history of smoking; primary tumors located in the cervical (n = 107, 9.5%), upper thoracic (n = 81, 7.9%), middle thoracic (n = 347, 34.0%), or lower thoracic region (n = 375, 36.7%), or in the abdominal esophagus (n = 217, 21.3%); combined chemotherapy and radiation therapy administered to 716 (8.9%); 333 (0.4%) underwent surgery combined with chemotherapy or radiation; and 78 (0.1%) underwent surgery alone. Surgical procedures included abdominocervical (transhiatal) esophagectomy (n = 281), Ivor-Lewis esophagectomy (n = 330), McKeown esophagectomy (n = 76), lower esophagectomy with gastrectomy (n = 291), and open esophageal stent placement (n = 149).

## Discussion

The prevalence and histological types of EC among different regions and countries varies widely and is explained by differences in carcinogenic factors that cause squamous cell carcinoma and adenocarcinoma of the esophagus (Faiz et al., 2019; Nassri et al, 2018; Wong et al., 2018). Uzbekistan is a high-risk area for EC (Asombang et al,, 2016; Hamrah et al, 2014). However, there is no comprehensive report on the dynamics of EC in Uzbekistan. We therefore investigated the transition in trends of EC in Uzbekistan from 2000 to 2018. This is the first study to summarize the epidemiological, histological, and clinical characteristics of EC in Uzbekistan.

During the study period (2000–2018), the mean incidence of EC was 2.8 per 100,000. Of note, the trend turned downward after 2010. These data are consistent with GLOBOCAN data published by the International Agency for Research on Cancer (Bray et al, 2018; Wong et al, 2018). The incidence of EC per 100,000 persons was lower in Uzbekistan compared with countries in East Asia (e.g. 30.8 for men and 5.6 for women in 2014 in Japan). There is a possibility that these rates may decrease, if the diagnostic accuracy, consultation rate, and cancer screening are improved.

We show here the distribution of age upon diagnosis. For example, the incidence of EC (37.5%) of patients aged 61 years to 70 years was highest, and few patients were 20 years or less or 81 years or more. This trend is similar to those of other Asian countries (Bray et al, 2018; Wong et al, 2018). Identification of a peak age of onset may contribute to establishing a cancer screening system by enhancing cost-to-benefit performance. The proportion of older patients with EC patients may increase, because life expectancy has increased (Kanda et al., 2019b).

Similar to East Asian countries, squamous cell carcinoma was the predominant tumor phenotype of patients with EC in Uzbekistan (Hong et al., 2015; Lagergren et al, 2017). Interestingly, there is a recent increasing trend in the incidence of adenocarcinoma. Future studies should therefore focus on the differences in daily activities, physical attributes, and genetic backgrounds of patients in East Asia to better understand the significance of trends in histological types of EC in Uzbekistan.

Our present findings show that little patients were diagnosed during the early stage of EC in Uzbekistan. We speculate that most patients visited a clinic and underwent endoscopy if they exhibited overt symptoms such as dysphagia and chest pain. Stage III EC, which was the most frequent stage among our subjects, confers a high risk of developing a recurrence and likely accounts for 5-year survival rates that plateaued at 25% (Hong et al, 2015; van Laarhoven et al., 2017). These findings indicate that reducing tumor progression to stage III will improve outcomes of EC in Uzbekistan.

The centralizated system established in Uzbekistan to treat EC may have a decided advantage. Among 17,144 patients registered, approximately half were treated at the RSSPMCO&R. A centralized system will likely be effective for standardizing treatment, implementing multidisciplinary therapy as well as improving surgical quality and postoperative follow-up. Moreover, enhanced collaboration among community clinics will be implemented to improve early detection rates and postoperative follow-up programs.

Several limitations of the present study should be acknowledged. The data obtained from our database comprise the official reports of regional clinics, and, therefore, the accumulation of additional data is difficult. Furthermore, no detailed data for environmental and genetic risk factors of EC was collected. Establishment of therapeutic systems that employ available treatment guidelines for perioperative treatment and surveillance programs, according to evidence acquired from Western and East Asian countries, will help develop a sophisticated database of prospective studies.

In conclusion, our review of the changes in the trends of EC in Uzbekistan over the past 20 years reveals that the incidence of EC gradually decreased, although the incidence of adenocarcinoma recently increased. Early detection to reduce the proportion of stage III EC is the most important consideration to improve prognosis.
